# Supplementary material for: Potential of Staphylea holocarpa Wood for Renewable Bioenergy
Source: Molecules. 2022 Dec 30;28(1):299. doi: 10.3390/molecules28010299 (PMC9822470; doi:10.3390/molecules28010299)
Supplement: Supplementary file 1 [file molecules-28-00299-s001.zip › molecules-2099583-SI.pdf]

This file includes:

Table S1-S5

Tables

Table S1 GC-MS analysis of ethanol sample.

No.	Retention Time (min)	Peak Area (%)	Component
1	5.30	4.99	Furfural
2	5.92	1.95	Dihydroxyacetone
3	8.79	5.29	D-Alanine, N-propargyloxycarbonyl-, isohexyl ester
4	9.91	6.09	4H-Pyran-4-one, 2,3-dihydro-3,5-dihydroxy-6-methyl-
5	11.30	24.66	5-Hydroxymethylfurfural
6	12.09	5.47	Butyl 2-acetoxyacetate
7	12.27	2.14	4H-Pyran-4-one, 2,3-dihydro-3,5-dihydroxy-6-methyl-
8	13.88	2.36	4-Nonanol
9	15.64	4.91	Sucrose
10	24.08	5.84	Acetic acid, 4,4-dimethylcyclopent-1-en-3-one-1-yl, (-)menthyl esterTER
11	28.41	1.52	Cyclotetrasiloxane, octamethyl-
12	29.48	20.75	dl-.alpha.-Tocopherol
13	32.48	2.91	Cyclotetrasiloxane, octamethyl-
14	33.59	8.33	Cyclotrisiloxane, hexamethyl-
15	33.83	2.79	Cyclotrisiloxane, hexamethyl-

Table S2 GC-MS analysis of the methanol sample.

No.	Retention Time (min)	Peak Area (%)	Component
1	5.43	1.37	2-Furanmethanol
2	5.58	0.98	3-Furanmethanol
3	5.73	1.77	Propanoic acid, 3-nitro-, methyl ester
4	5.95	8.88	Dihydroxyacetone
5	7.11	1.83	4H-Pyran-4-one, 2,3-dihydro-3,5-dihydroxy-6-methyl-
6	8.24	1.07	DL-Arabinose
7	8.59	0.46	Butanedioic acid, methylene-
8	8.88	9.36	D-Alanine, N-propargyloxycarbonyl-, isohexyl ester
9	9.79	0.51	l-Alanine, N-methoxycarbonyl-, butyl ester
10	9.96	7.88	4H-Pyran-4-one, 2,3-dihydro-3,5-dihydroxy-6-methyl-
11	10.16	4.40	L-Sorbose
12	10.65	0.56	Isosorbide Dinitrate
13	11.14	1.01	2-Deoxy-D-galactose

14	11.36	20.26	5-Hydroxymethylfurfural
15	11.63	6.55	1,2,3-Propanetriol, 1-acetate
16	12.23	2.84	D-Galactose
17	12.39	1.13	Lactose
18	12.79	1.39	Ethanone, 1-(2-hydroxy-5-methylphenyl)-
19	12.88	1.34	Pentandioic acid, (p-t-butylphenyl) ester
20	14.14	0.56	2-Formyl-4-methylpentanoic acid, ethyl ester
21	15.80	2.04	D-Allose
22	16.72	0.64	1-Cyclopentene, 3-methylene-1-trimethylsilyloxy-
23	17.99	1.06	3-Deoxy-d-mannoic lactone
24	18.68	0.61	d-Gulopyranose
25	19.15	0.50	Benzeneacetic acid, 4-hydroxy-3-methoxy-, methyl ester
26	19.27	1.93	4-Hydroxy-2-methoxycinnamaldehyde
27	21.91	1.34	n-Hexadecanoic acid
28	22.46	0.67	3,5-Dimethoxy-4-hydroxycinnamaldehyde
29	24.10	3.12	Linoelaidic acid
30	24.19	1.68	(R)-(-)-14-Methyl-8-hexadecyn-1-ol
31	29.45	0.85	Cyclotrisiloxane, hexamethyl-
32	30.04	4.95	Cyclotetrasiloxane, octamethyl-
33	30.92	0.26	Cyclotetrasiloxane, octamethyl-
34	31.49	0.49	Cyclotetrasiloxane, octamethyl-
35	32.87	0.61	Cyclotetrasiloxane, octamethyl-
36	33.36	1.32	Cyclotetrasiloxane, octamethyl-
37	33.61	3.80	Cyclotetrasiloxane, octamethyl-

Table S3 GC-MS analysis of benzene/ethanol sample

No.	Retention Time (min)	Peak Area (%)	Component
1	5.18	0.51	Glyceraldehyde
2	5.99	1.30	Dihydroxyacetone
3	7.88	47.63	1-Hexanol, 2-ethyl-
4	8.82	2.82	D-Alanine, N-propargyloxycarbonyl-, isohexyl ester
5	9.93	3.68	4H-Pyran-4-one, 2,3-dihydro-3,5-dihydroxy-6-methyl-
6	11.32	7.60	5-Hydroxymethylfurfural
7	11.56	0.73	1,2,3-Propanetriol, 1-acetate
8	12.14	4.10	Butyl 2-acetoxyacetate
9	12.33	1.45	4H-Pyran-4-one, 2,3-dihydro-3,5-dihydroxy-6-methyl-
10	13.94	1.03	Acetoxyacetic acid, nonyl ester
11	19.29	3.94	4-((1E)-3-Hydroxy-1-propenyl)-2-methoxyphenol
12	20.97	0.56	2-Propenoic acid, 3-(4-hydroxy-3-methoxyphenyl)-

13	21.94	1.24	n-Hexadecanoic acid
14	22.12	4.72	Dibutyl phthalate
15	22.30	0.51	1-Hexyl-1-nitrocyclohexane
16	24.11	3.11	Linoelaidic acid
17	24.20	1.73	Methoxyacetic acid, dodec-9-ynyl ester
18	27.17	0.99	Ethanol
19	27.83	0.83	Ethanol
20	28.47	0.69	Ethanol
21	29.09	0.70	Ethanol
22	29.34	2.46	Phthalic acid, di(6-methylhept-2-yl) ester
23	29.70	0.65	Ethanol
24	32.31	1.08	Cyclotetrasiloxane, octamethyl-
25	32.52	1.12	Cyclotetrasiloxane, octamethyl-
26	32.88	2.17	Cyclotetrasiloxane, octamethyl-
27	33.38	2.65	Cyclotetrasiloxane, octamethyl-

Table S4 GC-MS analysis of the ethanol/methanol sample.

No.	Retention Time (min)	Peak Area (%)	Component
1	5.37	3.82	Furfural
2	5.59	1.48	2-Furanmethanol
3	5.75	0.67	l-Alanine, N-methoxycarbonyl-, butyl ester
4	5.98	1.73	Dihydroxyacetone
5	7.12	0.40	4H-Pyran-4-one, 2,3-dihydro-3,5-dihydroxy-6-methyl-
6	8.27	0.55	Tetrahydro-4H-pyran-4-ol
7	8.93	3.38	D-Alanine, N-propargyloxycarbonyl-, isohexyl ester
8	10.01	3.96	4H-Pyran-4-one, 2,3-dihydro-3,5-dihydroxy-6-methyl-
9	10.54	0.63	2-Butanone, 4-hydroxy-3-methyl-
10	11.67	30.93	5-Hydroxymethylfurfural
11	11.83	1.83	5-Hydroxymethylfurfural
12	12.39	1.00	Acetoxyacetic acid, nonyl ester
13	12.82	3.04	Ethanone, 1-(2-hydroxy-5-methylphenyl)-
14	13.04	1.16	1,6:3,4-Dianhydro-2-O-acetyl-.beta.-d-talopyranose
15	14.18	0.89	1,4-Dioxane, 2-ethyl-5-methyl-
16	14.26	0.38	4-Nonanol
17	14.83	0.39	di-n-Propylmalonic acid
18	15.00	0.61	Phenol, 2-methoxy-4-(1-propenyl)-, (Z)-
19	15.73	0.73	.alpha.-D-Glucose
20	16.17	2.38	.beta.-D-Glucopyranose, 1,6-anhydro-
21	16.33	0.97	.beta.-D-Glucopyranose, 1,6-anhydro-

22	17.90	0.69	1,6-Anhydro-.beta.-D-glucofuranose
23	18.06	0.39	Homovanillic acid
24	18.72	0.45	2-Butanone, 4-(4-hydroxy-3-methoxyphenyl)-
25	19.23	0.40	2-Propanone, 1-hydroxy-3-(4-hydroxy-3-methoxyphenyl)-
26	19.32	1.43	4-Hydroxy-2-methoxycinnamaldehyde
27	20.66	0.37	11-Bromo-1-undecanol, TMS derivative
28	21.16	2.14	2-Propenoic acid, 3-(4-hydroxy-3-methoxyphenyl)-
29	22.00	2.23	n-Hexadecanoic acid
30	22.13	0.48	Dibutyl phthalate
31	22.58	2.98	10.alpha.-Eremophilane
32	24.18	3.86	Linoelaidic acid
33	24.25	0.56	Chloroacetic acid, dodec-9-ynyl ester
34	24.30	0.53	Chloroacetic acid, dodec-9-ynyl ester
35	29.33	0.69	2(3H)-Furanone, dihydro-3,4-bis[(4-hydroxy-3-methoxyphenyl)methyl]-, (3R-trans)-
36	29.61	0.51	.alpha.-Tocopheryl acetate
37	30.16	0.52	1,4-Bis(trimethylsilyl)benzene
38	30.85	4.68	1,4-Bis(trimethylsilyl)benzene
39	31.03	1.56	Benzimidazole, 2-benzylsulfonyl-
40	31.32	3.40	1,4-Bis(trimethylsilyl)benzene
41	31.95	4.21	Arsenous acid, tris(trimethylsilyl) ester
42	32.46	5.64	Arsenous acid, tris(trimethylsilyl) ester
43	32.90	0.69	1,2-Bis(trimethylsilyl)benzene
44	33.54	0.64	Cyclotetrasiloxane, octamethyl-

Table S5 Py-GC-MS analysis of *S. holocarpa* wood.

No.	Retention Time (min)	Peak Area (%)	Component
1	3.70	0.020	Cyclobutanol
2	4.10	5.769	Ethyne, fluoro-
3	4.26	2.785	Acetaldehyde
4	4.67	1.463	Methyl glyoxal
5	4.94	0.722	Formic acid
6	5.15	0.012	Formic acid
7	5.24	0.352	Acetaldehyde, hydroxy-
8	5.38	1.431	Acetaldehyde, hydroxy-
9	5.48	0.906	2,3-Butanedione
10	5.62	0.230	Acetic acid, hydroxy-

11	5.71	0.092	Methyl formate
12	5.83	0.068	Hydrazine, ethyl-
13	6.64	0.215	2-Propanone, 1-hydroxy-
14	6.75	2.523	2-Propanone, 1-hydroxy-
15	6.86	0.157	1-Propanol, 2-methyl-
16	6.93	0.078	Acetic acid, ethoxy-
17	7.00	0.132	Ethyl formate
18	7.11	0.062	Methyl formate
19	7.23	0.107	2,3-Pentanedione
20	7.43	0.081	Acetic acid, hydroxy-, methyl ester
21	7.53	0.392	3-Pentanone
22	7.62	0.042	Ethyl formate
23	7.69	0.044	Ethyl ether
24	7.77	0.091	Ethyl ether
25	7.84	0.174	Ethyl ether
26	7.93	0.148	1,2-Ethanediol, monoformate
27	8.02	0.125	1,2-Ethanediol
28	8.36	0.059	Ethyl formate
29	8.40	0.020	Dihydroxyacetone
30	8.45	0.049	Glycolaldehyde dimer
31	8.63	0.332	2-Butenal, 2-methyl-
32	8.69	0.195	2-Propenoic acid, 2-hydroxyethyl ester
33	8.75	0.088	2,3-Diazabicyclo[2.2.1]-hept-2-ene
34	9.15	1.966	Acetic acid, methyl ester
35	9.29	0.489	Acetic acid, 2-ethylbutyl ester
36	9.41	0.053	Acetic acid, hydrazide
37	9.54	1.437	Succindialdehyde
38	9.64	0.337	Propanoic acid, 2-oxo-, methyl ester
39	9.73	1.365	Propanoic acid, 2-oxo-, methyl ester
40	9.79	0.126	Oxirane, (butoxymethyl)-
41	9.92	0.361	Glycidol
42	10.02	0.362	N'-(Diaminomethylidene)formohydrazide
43	10.30	0.072	Acetone, ethyl methyl acetal
44	10.41	0.177	Furfural
45	10.62	0.126	2-Furanmethanol
46	10.98	0.080	2-Ethylideneamino-propionitrile
47	11.07	1.183	Furfural
48	11.13	0.348	2-Cyclopenten-1-one
49	11.35	0.097	N-Acrylonitrylaziridine
50	11.45	0.142	CH_2=C(CH3)CH_2COOH

51	11.72	0.077	2H-Pyran, 3,4-dihydro-6-methyl-
52	11.82	0.930	1,6:2,3-Dianhydro-4-O-acetyl-.beta.-d-mannopyranose
53	12.01	0.086	1-Hydroxy-2-butanone
54	12.14	0.398	2-Propanone, 1-(acetyloxy)-
55	12.22	0.163	2(5H)-Furanone, 5-methyl-
56	12.35	0.036	5H-1,4-Dioxepin, 2,3-dihydro-
57	12.51	0.037	4-Cyclopentene-1,3-dione
58	12.63	0.018	1-Buten-3-yne, 2-methyl-
59	12.74	0.214	Cyclopent-4-ene-1,3-dione
60	13.37	0.232	2-Butenoic acid, methyl ester, (E)-
61	13.48	0.120	2-Cyclopenten-1-one, 2-methyl-
62	13.61	0.122	Furan, 2-ethyl-5-methyl-
63	13.84	1.015	2(5H)-Furanone
64	13.96	0.140	Ethanol, 2-methoxy-, carbonate (2:1)
65	14.27	2.272	2-Cyclopenten-1-one, 2-hydroxy-
66	14.65	0.213	2,5-Furandione, 3-methyl-
67	14.84	0.068	Cyclohexane, methyl-
68	15.02	0.075	7-Oxabicyclo[4.1.0]heptane, 1-methyl-
69	15.16	0.048	4,5-Dihydroxy-6-hydroxymethyl-oxepan-3-one
70	15.21	0.109	4,5-Diethyl-3,6-octandione
71	15.35	0.409	2-Furancarboxaldehyde, 5-methyl-
72	15.48	0.195	2-Cyclopenten-1-one, 3-methyl-
73	15.72	0.049	Furyl hydroxymethyl ketone
74	15.85	0.123	1H-Imidazole-2-carboxaldehyde, 1-methyl-
75	15.91	0.270	Phenol
76	16.38	0.044	2,4-Hexadiene, 2,5-dimethyl-
77	16.52	0.255	2H-Pyran-2,6(3H)-dione
78	16.61	0.857	2-Methyliminoperhydro-1,3-oxazine
79	16.77	0.091	3-Amino-2-oxazolidinone
80	16.83	0.119	9-Azabicyclo[6.1.0]non-4-en-9-amine, (1.alpha.,4Z,8.alpha.)-
81	17.19	0.225	1H-Tetrazaborole, 4,5-dihydro-1,4,5-trimethyl-
82	17.33	0.048	2-Propenal, 3-(dimethylamino)-
83	17.46	0.029	d-Mannitol, 1,4-anhydro-
84	17.65	1.049	1,2-Cyclopentanedione, 3-methyl-
85	17.76	0.060	Ethanol, 2-[(1-methylene-2-propenyl)oxy]-
86	17.91	0.138	2-Cyclopenten-1-one, 2,3-dimethyl-
87	18.04	0.016	Benzaldehyde, 2-hydroxy-
88	18.12	0.145	4-Methyl-5H-furan-2-one
89	18.21	0.034	1,2,3-Oxadiazolium, 3-(2,2-dimethylbutyl)-5-hydroxy-, hydroxide, inner salt

90	18.30	0.132	Phenol, 2-methyl-
91	18.48	0.156	2-Cyclopenten-1-one, 2-hydroxy-3,4-dimethyl-
92	18.59	0.156	2H-Pyran-2-methanol, tetrahydro-
93	18.74	0.730	Furfuryl alcohol, tetrahydro-5-methyl-, cis-
94	18.91	0.267	p-Cresol
95	19.06	0.131	2-Methyl-2-vinyloxirane
96	19.18	0.043	Valeraldehyde, 2,2-dimethyl-, oxime
97	19.29	0.110	Furyl hydroxymethyl ketone
98	19.34	0.041	4-Formyl-1,3(2H)-dihydroimidazole-2-thione
99	19.44	1.668	Phenol, 2-methoxy-
100	19.56	0.062	Z-(13,14-Epoxy)tetradec-11-en-1-ol acetate
101	19.78	1.642	2(3H)-Furanone, dihydro-4-hydroxy-
102	19.99	0.015	2-Acetamido-2,3-dideoxy-d-glucose
103	20.20	0.124	Maltol
104	20.30	0.175	2-Cyclopenten-1-one, 3-ethyl-2-hydroxy-
105	20.48	0.509	2,4(3H,5H)-Furandione, 3-methyl-
106	20.68	0.041	2H-Pyran-2-one, 4-hydroxy-6-methyl-
107	20.76	0.014	6-Hydroxy-2-pyridinecarboxylic acid
108	20.82	0.064	1,3-Pentadiene
109	20.89	0.084	Phenol, 2,4-dimethyl-
110	21.12	0.328	Cyclopentanone, 2-methyl-
111	21.34	0.442	Oxalic acid, 4-chlorophenyl octyl ester
112	21.47	0.298	2,3-Dihydroxybenzaldehyde
113	21.62	0.055	Phenylephrine
114	21.70	0.192	Creosol
115	21.87	0.178	Creosol
116	22.03	1.648	Creosol
117	22.28	1.333	Catechol
118	22.58	0.454	1,4:3,6-Dianhydro-.alpha.-d-glucopyranose
119	22.71	0.131	4H-Pyran-4-one, 2,6-dimethyl-
120	22.78	0.198	Tetradecane, 4-methyl-
121	22.91	0.941	5-Hydroxymethylfurfural
122	23.07	0.096	Hexadecanenitrile
123	23.14	0.082	Oxacyclotridecan-2-one
124	23.22	0.152	trans-2-Dodecen-1-ol, trifluoroacetate
125	23.39	0.215	E-8-Methyl-9-tetradecen-1-ol acetate
126	23.48	0.317	1,2-Benzenediol, 3-methyl-
127	23.59	0.789	1,2-Benzenediol, 3-methoxy-
128	23.80	0.747	Phenol, 4-ethyl-2-methoxy-
129	23.84	0.229	Benzenethiol, o-isopropyl-,

130	23.90	0.121	2,2-Dimethyl-1-aza-spiro[2.4]heptane
131	23.96	0.211	Boranamine, 1,1-diethyl-N-phenyl-
132	24.08	0.467	1,2-Benzenediol, 4-methyl-
133	24.16	0.160	6-Hepten-1-ol, 2-methyl-
134	24.23	0.179	2-(2-Methyloxyryl)ethoxycarbonylmethoxyiminomethane
135	24.28	0.172	1,3,5-Benzenetriol
136	24.36	0.273	Benzaldehyde, 4-hydroxy-
137	24.48	2.747	2-Methoxy-4-vinylphenol
138	24.73	0.142	5,9-Dimethyl-2-(1-methylethylidene)-1-cyclodecanol
139	24.81	0.243	13-Methyltetradecanal
140	24.86	0.192	1,1-Dimethyl-5-trimethylsilyl-1-silacyclohex-3-ene
141	24.91	0.207	Methyl 6,8-dodecadienyl ether
142	25.11	2.854	Phenol, 2,6-dimethoxy-
143	25.20	0.826	Phenol, 2-methoxy-3-(2-propenyl)-
144	25.35	0.828	Phenol, 2-methoxy-4-propyl-
145	25.45	0.277	Methyl 6,8-dodecadienyl ether
146	25.61	0.188	2-(1-Methylcyclopropyl)thiophene
147	25.71	0.429	13-Octadecenal, (Z)-
148	25.84	0.467	(-)-8-p-Menthen-2-yl, acetate, trans
149	25.96	1.063	Vanillin
150	26.01	0.940	Phenol, 2-methoxy-4-(1-propenyl)-
151	26.25	0.550	7-Oxabicyclo[4.1.0]hept-4-en-3-one, 1,2,2,4,5,6-hexamethyl-
152	26.46	0.200	(-)-Spathulenol
153	26.60	1.490	3,5-Dimethoxy-4-hydroxytoluene
154	26.68	2.183	trans-Isoeugenol
155	26.86	0.496	Phenol, 2-methoxy-4-propyl-
156	26.94	0.148	Ketone, methyl 2-methyl-1-cyclohexen-1-yl, semicarbazone
157	27.00	0.172	d-Glucosamine, diethylmercaptal
158	27.09	0.534	Nonanoic acid
159	27.21	0.338	Benzene, hexamethyl-
160	27.33	0.690	Apocynin
161	27.45	0.458	Benzene, 4-ethyl-1,2-dimethoxy-
162	27.59	0.131	Spiro[4.5]decan-7-one, 1,8-dimethyl-8,9-epoxy-4-isopropyl-
163	27.63	0.186	1H-6-Purinone,6,7-dihydro-2-amino-7-[3,5-dihydroxy-6-(hydroxy methyl)tetrahydro-2H-2-pyranyl]
164	27.75	0.536	.beta.-D-Glucopyranose, 1,6-anhydro-
165	27.83	0.983	5-tert-Butylpyrogallol
166	28.03	2.797	2-Propanone, 1-(4-hydroxy-3-methoxyphenyl)-
167	28.13	0.817	D-Allose
168	28.20	1.000	.beta.-D-Glucopyranose, 1,6-anhydro-

169	28.39	0.603	Nonadecanoic acid
170	28.54	2.942	(E)-Stilbene
171	28.77	0.657	4-(1-Hydroxyallyl)-2-methoxyphenol
172	28.91	0.320	1-(3,4-methylenedioxyphenyl)propane-1-ol
173	28.97	0.181	Benzoic acid, 4-hydroxy-3-methoxy-, methyl ester
174	29.05	0.221	.beta.-D-Glucopyranoside, methyl 4,6-O-nonylidene-
175	29.15	0.716	Phenol, 2,6-dimethoxy-4-(2-propenyl)-
176	29.27	0.559	2-Propanone, 1,1-diphenyl-
177	29.68	0.164	Octadecane, 1-bromo-
178	29.76	0.120	5-Isopropenyloxymethylene-3,3-dimethyl-cyclohexanone
179	29.85	0.257	Propenoic acid, 3-(bicyclo[2.2.1]hept-1-yl)-, methyl ester
180	30.10	0.841	(E)-2,6-Dimethoxy-4-(prop-1-en-1-yl)phenol
181	30.17	0.963	Benzenepropanol, 4-hydroxy-3-methoxy-
182	30.40	0.886	Benzaldehyde, 4-hydroxy-3,5-dimethoxy-
183	30.66	0.846	4-((1E)-3-Hydroxy-1-propenyl)-2-methoxyphenol
184	30.88	0.209	cis-2-Methyl-4-phenylthiane
185	31.04	0.174	3-(1-Methyl-1-silacyclobutyl)benzoic acid, methyl ester
186	31.19	2.050	(E)-2,6-Dimethoxy-4-(prop-1-en-1-yl)phenol
187	31.29	0.337	4-Propyl-1,1'-diphenyl
188	31.48	0.232	Estra-1,3,5(10)-trien-17.beta.-ol
189	31.72	0.140	4,6,10,10-Tetramethyl-5-oxatricyclo[4.4.0.0(1,4)]dec-2-en-7-ol
190	32.04	0.461	Ethanone, 1-(4-hydroxy-3,5-dimethoxyphenyl)-
191	32.19	0.985	Coniferyl aldehyde
192	32.26	1.525	4-((1E)-3-Hydroxy-1-propenyl)-2-methoxyphenol
193	32.96	0.755	1-Butanone, 1-(2,4,6-trihydroxy-3-methylphenyl)-
194	33.29	0.106	5H-Dibenzo[a,d]cyclohepten-5-ol, 10,11-dihydro-
195	33.39	0.035	Spiro[4.5]decan-7-one, 1,8-dimethyl-8,9-epoxy-4-isopropyl-
196	33.52	0.070	9-Hexadecenoic acid, methyl ester, (Z)-
197	33.77	0.014	2-Dodecen-1-yl(-)succinic anhydride
198	34.31	0.079	Silane, bromotriethyl-
199	34.51	0.162	4'-Phenylpropiophenone
200	34.62	0.045	4-Isothiazolecarbonitrile, 5-methyl-3-phenyl-
201	34.75	0.057	12-Methyl-E,E-2,13-octadecadien-1-ol
202	35.64	0.316	Docosanoic acid
203	35.76	0.087	(2,2-Bis(ethylthio)-1,2,3,4-tetrahydronaphthalen-1-yl)methanol
204	36.43	0.109	9-Undecenal, 2,10-dimethyl-
205	36.45	0.078	9-Undecenal, 2,10-dimethyl-
206	36.88	0.461	5-(3-Hydroxypropyl)-2,3-dimethoxyphenol
207	37.12	0.084	5-(Acetylaminomethyl)-4-amino-2-methylpyrimidine
208	37.31	0.084	Guanidine, N-[3-[(2-bromophenyl)amino]-1-propenyl]-

209	37.83	0.369	trans-Sinapyl alcohol
210	37.99	0.049	1,4-Pentadien-3-one, 1,5-diphenyl-
211	39.16	0.498	n-Hexadecanoic acid
212	39.42	0.076	Hexadec-2-enylsuccinic anhydride
213	39.67	0.090	Phthalic acid, hex-2-yn-4-yl isohexyl ester
214	40.27	0.103	Thioguanine
